# Supplementary material for: PGAM5 cleavage and oligomerization equilibrates mitochondrial dynamics under stress by regulating DRP1 function
Source: J Cell Sci. 2025 Nov 14;138(21):jcs263903. doi: 10.1242/jcs.263903 (PMC12669963; doi:10.1242/jcs.263903)
Supplement: Supplementary information [file joces-138-263903-s1.pdf]

Supplementary Fig 1

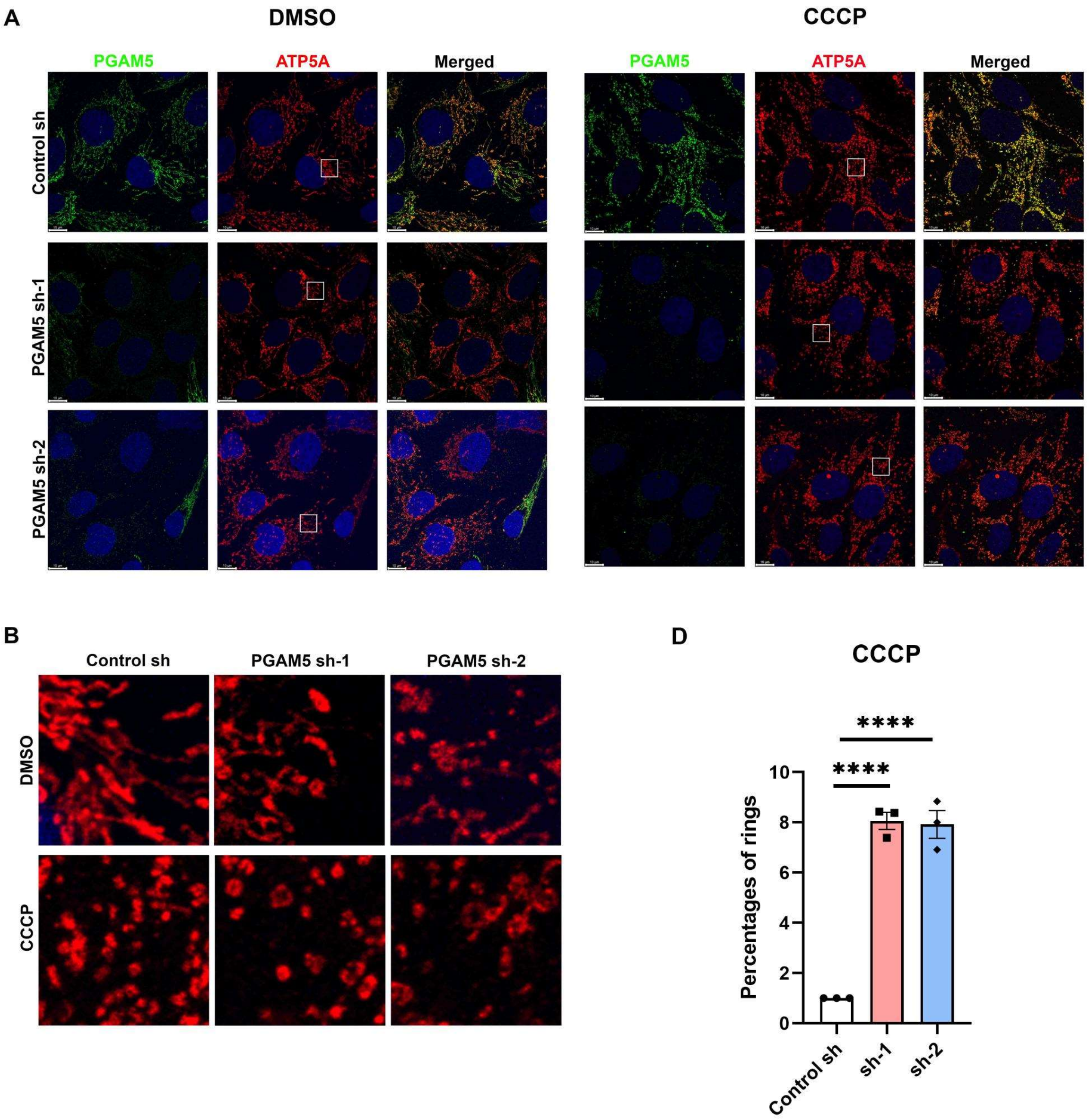

Fig. S1.

A. Confocal images of U2OS cells stably expressing Control sh or PGAM5 sh-1 or PGAM5 sh-2 under DMSO and CCCP (20 µM for 2hrs) treated conditions. Scale bars 10 µm.
B. Zoomed-in insets of the knockdown phenotype. The zoomed-in region is marked with the white box. Scale bars 5 µm.
C. The bar diagram showing the comparison of quantification of the ring structures between control and PGAM5 KD U2OS cells under CCCP-treated condition. Values are presented as mean ± SEM (n=3). *p* value Control sh: sh-1 ****<0.0001; Control sh: sh-2 ****<0.0001 (One-way ANOVA with Tukey's multiple comparison test).

Journal of Cell Science • Supplementary information

Supplementary Fig 2

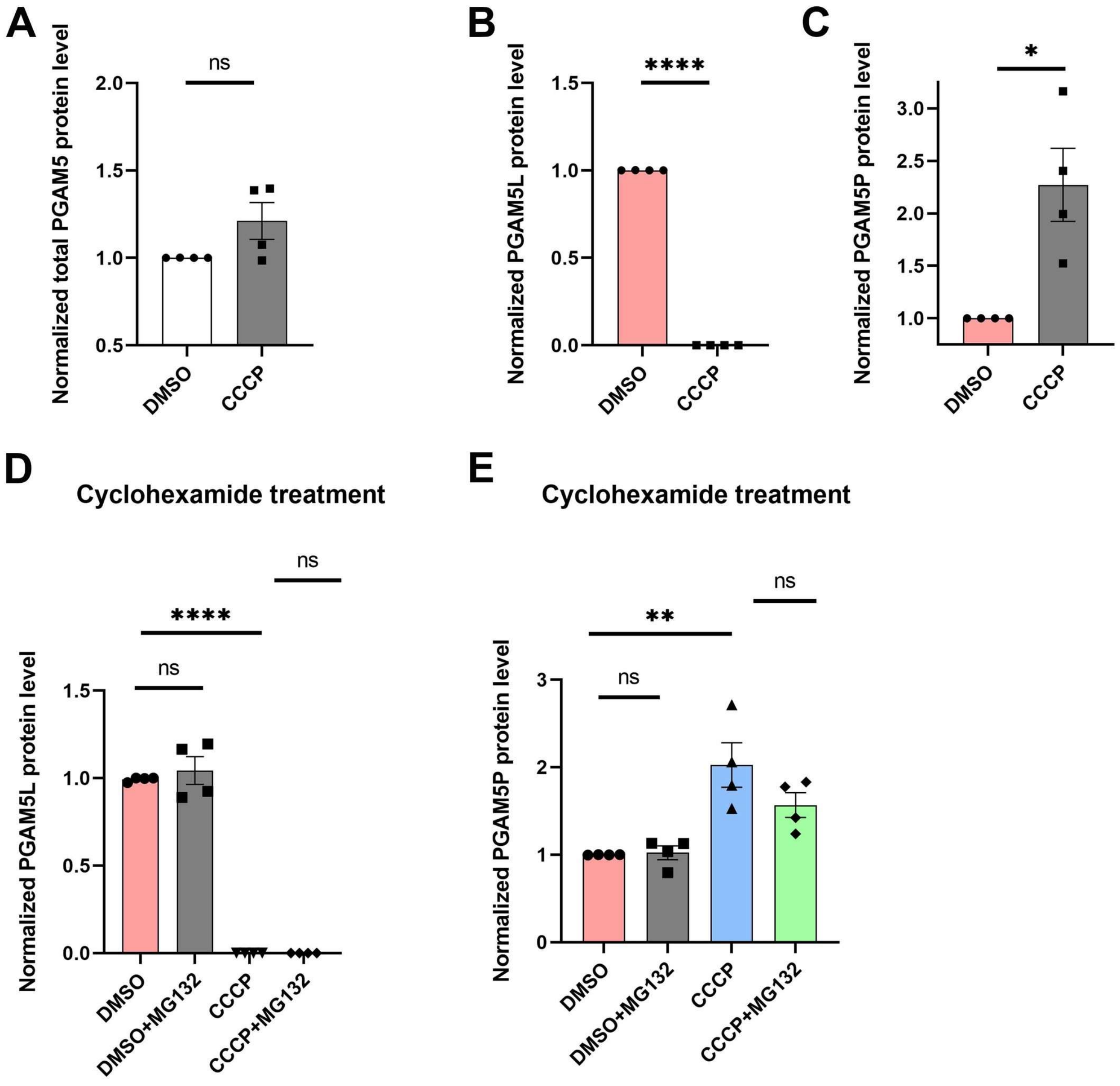

Journal of Cell Science • Supplementary information

Fig. S2.

A. The bar diagram shows the normalized total PGAM5 protein level under DMSO and CCCP treated conditions. Values are presented as mean ± SEM (n=4) . *p* 0.1398 (non-significant) (Paired t-test).

B. The bar diagram shows the normalized PGAM5L protein level under DMSO and CCCP treated conditions. Values are presented as mean ± SEM (n=4). *****p* <0.0001 (Paired t-test).

C. The bar diagram shows the normalized PGAM5P protein level under DMSO and CCCP treated conditions. Values are presented as mean ± SEM (n=4) using GraphPad Prism. **p* 0.0351 (Paired t-test).

D. The bar diagram shows the normalized PGAM5L protein level under various treatment conditions. means ± SEM (n = 4). *p* value DMSO: DMSO+MG132: 0.8076 (non-significant); DMSO: CCCP ****<0.0001; DMSO+MG132: CCCP ****<0.0001; CCCP: CCCP+MG132 >0.9999(non-significant) (One-way ANOVA with Tukey's multiple comparison test).

E. The bar diagram shows the normalized PGAM5P protein level under various treatment conditions. means ± SEM (n = 4). *p* value DMSO: DMSO+MG132: 0.9995 (non-significant); DMSO: CCCP **0.0021; DMSO+MG132: CCCP **0.0025; CCCP: CCCP+MG132 0.1942(non-significant) (One-way ANOVA with Tukey's multiple comparison test).

Journal of Cell Science • Supplementary information

Supplementary Fig 3

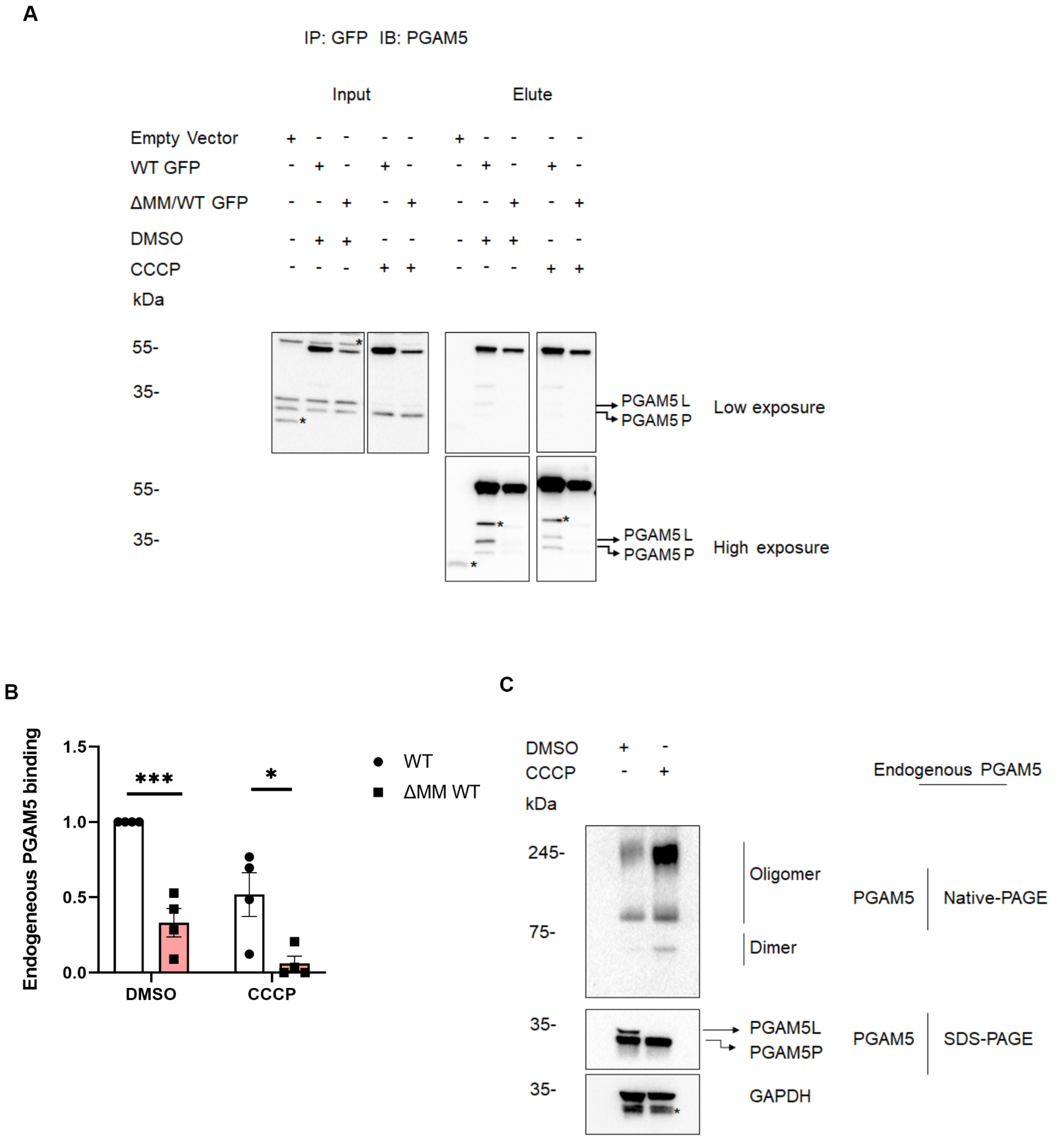

Journal of Cell Science • Supplementary information

Fig. S3.

A. Representative Western blots showing the interaction pattern between PGAM5 WT GFP or ΔMM WT GFP mutant expressed in HEK293 cells with endogenous PGAM5 under DMSO and CCCP-treated conditions. * marks the non-specific bands.

B. Endogenous PGAM5 binding was normalized to PGAM5 GFP pulldown and relative binding amount in DMSO and CCCP treated conditions were plotted. The bar graph shows the comparison of the relative endogenous PGAM5 binding between DMSO and CCCP -treated conditions. means ± SEM (n = 4). *p* value WT DMSO: ΔMM WT DMSO: ***0.0010; WT CCCP: ΔMM WT CCCP *0.165; WT DMSO: WT CCCP *0.0119; ΔMM WT DMSO: ΔMMWT CCCP 0.1984(non-significant) (Two-way ANOVA with Tukey's multiple comparison test).

C. Representative western blots showing the separation of same amount of PGAM5 protein from U2OS cells treated with DMSO and CCCP, in Native-PAGE and SDS-PAGE gels. * marks the non-specific bands.

Journal of Cell Science • Supplementary information

Supplementary Figure 4

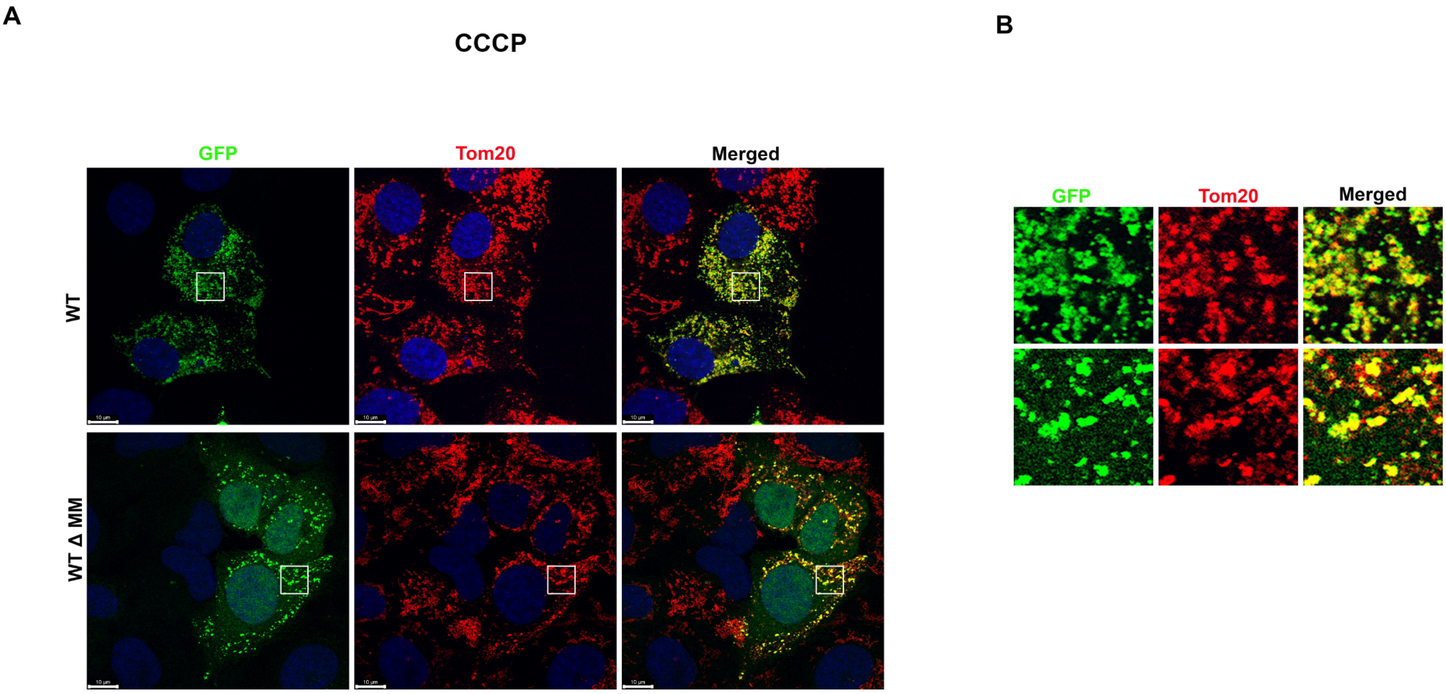

Fig. S4.

A. Confocal images of U2OS cells transiently transfected with either PGAM5 WT GFP or PGAM5 WT ΔMM GFP under CCCP (20 µM for 2hrs) treated condition. Scale bars 10 µm.

B. Zoomed-in insets of the over expression phenotype. The zoomed-in region is marked with the white box. Scale bars 5 µm.

Journal of Cell Science • Supplementary information

Supplementary Figure 5

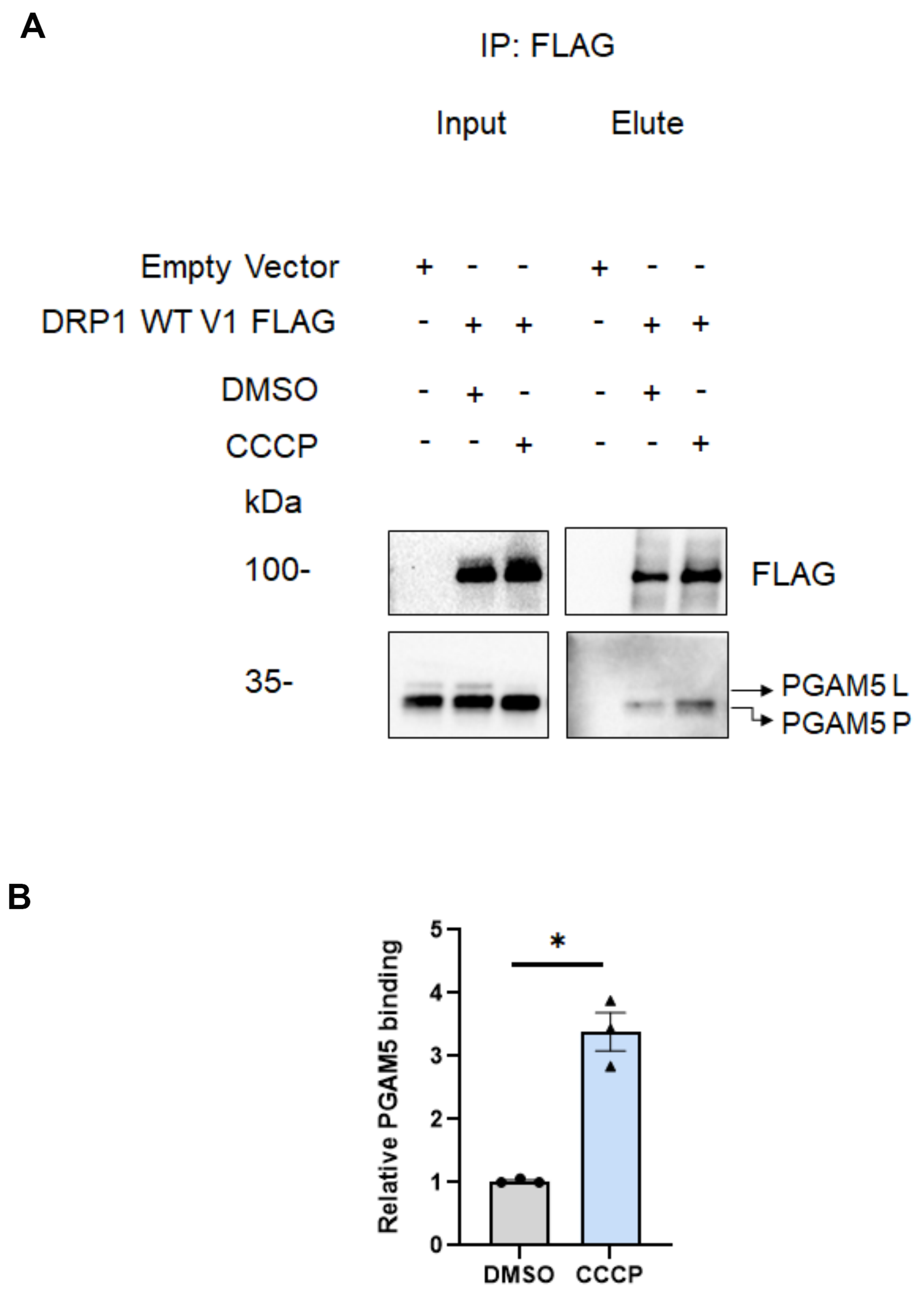

Fig. S5.

A. Representative Western blots showing the interaction pattern between DRP1 WT FLAG expressed in HEK293 cells with endogenous PGAM5 under DMSO and CCCP-treated conditions.
B. Endogenous PGAM5 was normalized to DRP1 WT FLAG pulldown and relative binding amount in DMSO and CCCP treated conditions were plotted. The bar graph shows the comparison of the relative endogenous PGAM5 binding between DMSO and CCCP -treated conditions. means ± SEM (n = 3). **p* value 0.0179 (Paired t-test).

Supplementary Fig 6

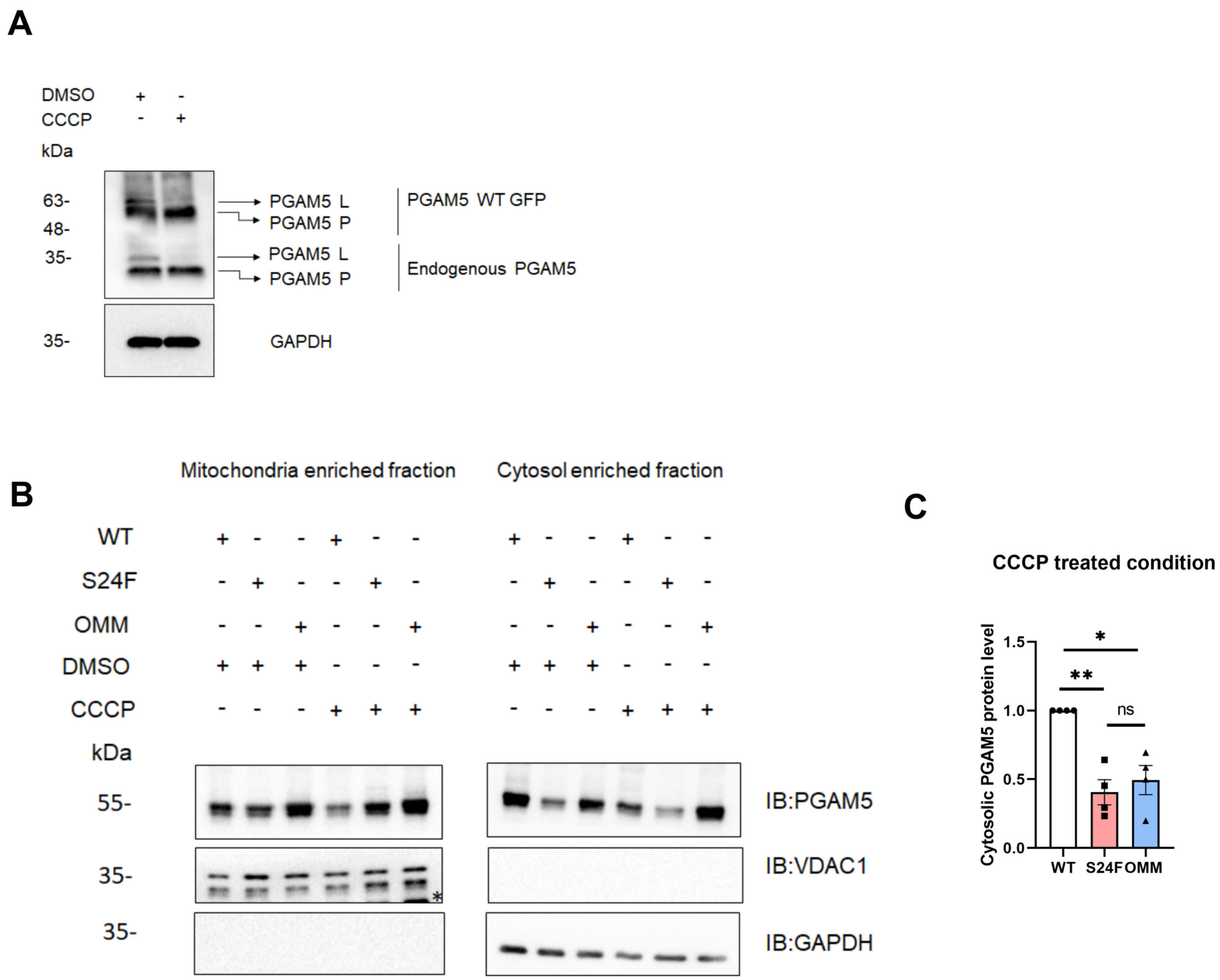

Fig. S6.

A. Representative Western blots from 4-20% gradient SDS PAGE showing the PGAM5 WT GFP cleavage pattern in transiently expressing U2OS cells treated with either DMSO or CCCP.

B. Representative Western blots showing the mitochondrial and cytosolic fractions of U2OS cells transiently expressing either PGAM5 WT/ S24F/ OMM mutant and treated with either DMSO or CCCP. * marks the non-specific band.

C. The bar diagram shows the normalized values of PGAM5 protein levels in the cytosolic fraction. means ± SEM (n = 4). *p* Value WT: S24F **0.0073, WT: OMM *0.0177, S24F: OMM 0.4598 (non-significant) (Paired t-test).

Journal of Cell Science • Supplementary information

Fig1A

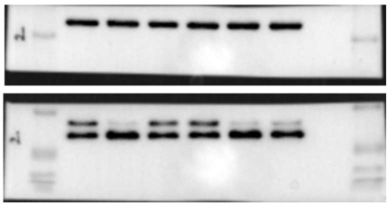

Fig1C

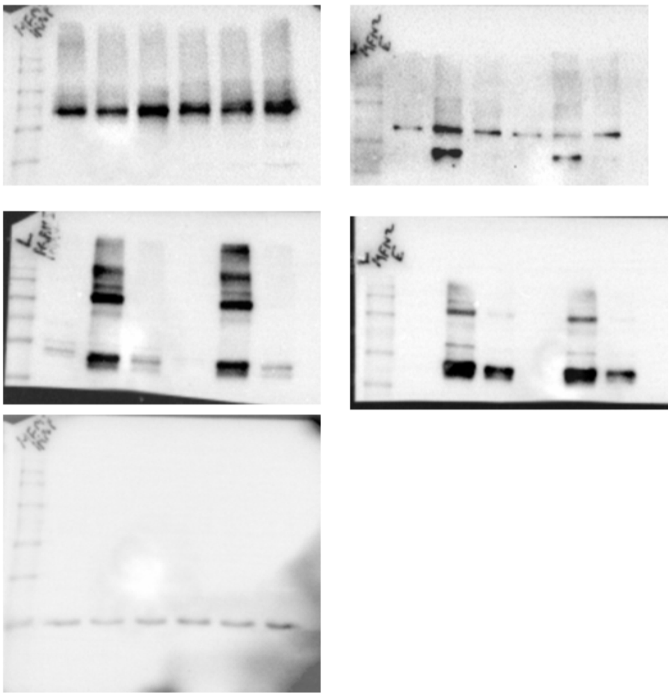

Fig1G

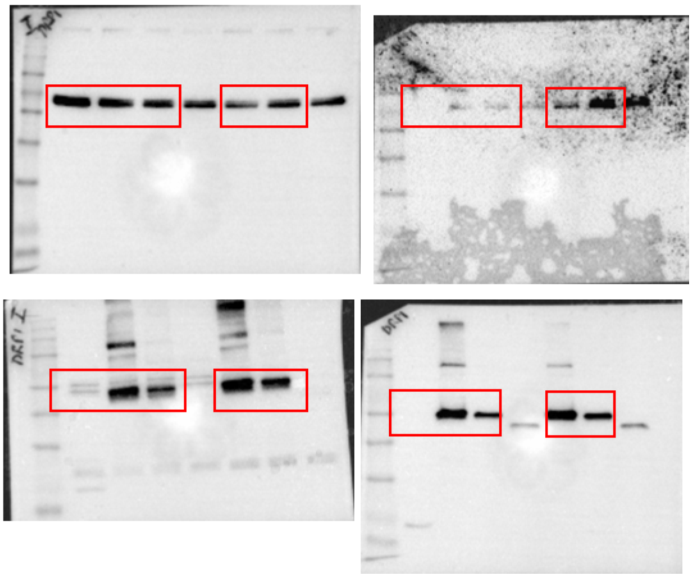

Fig 3B

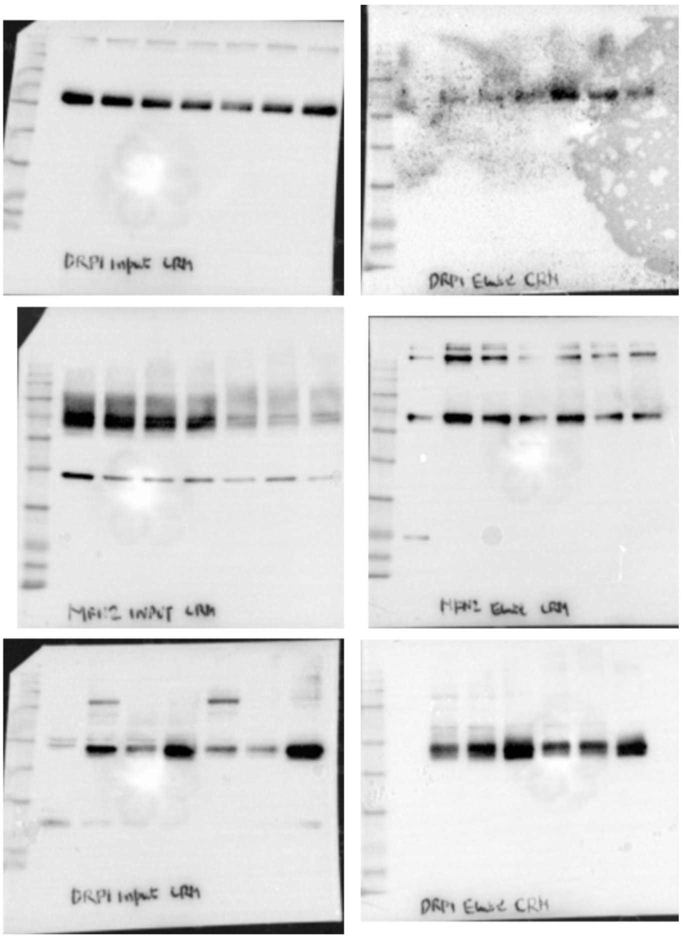

Journal of Cell Science • Supplementary information

Fig 4E

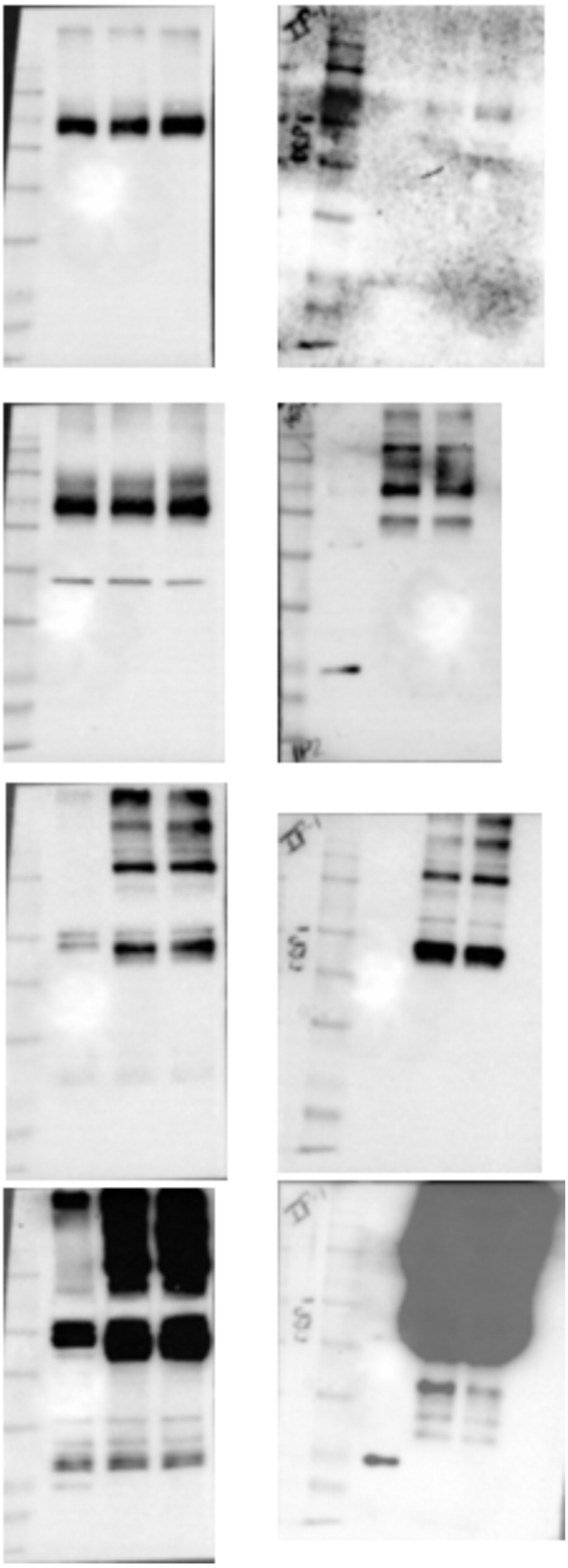

Journal of Cell Science • Supplementary information

Fig 5A

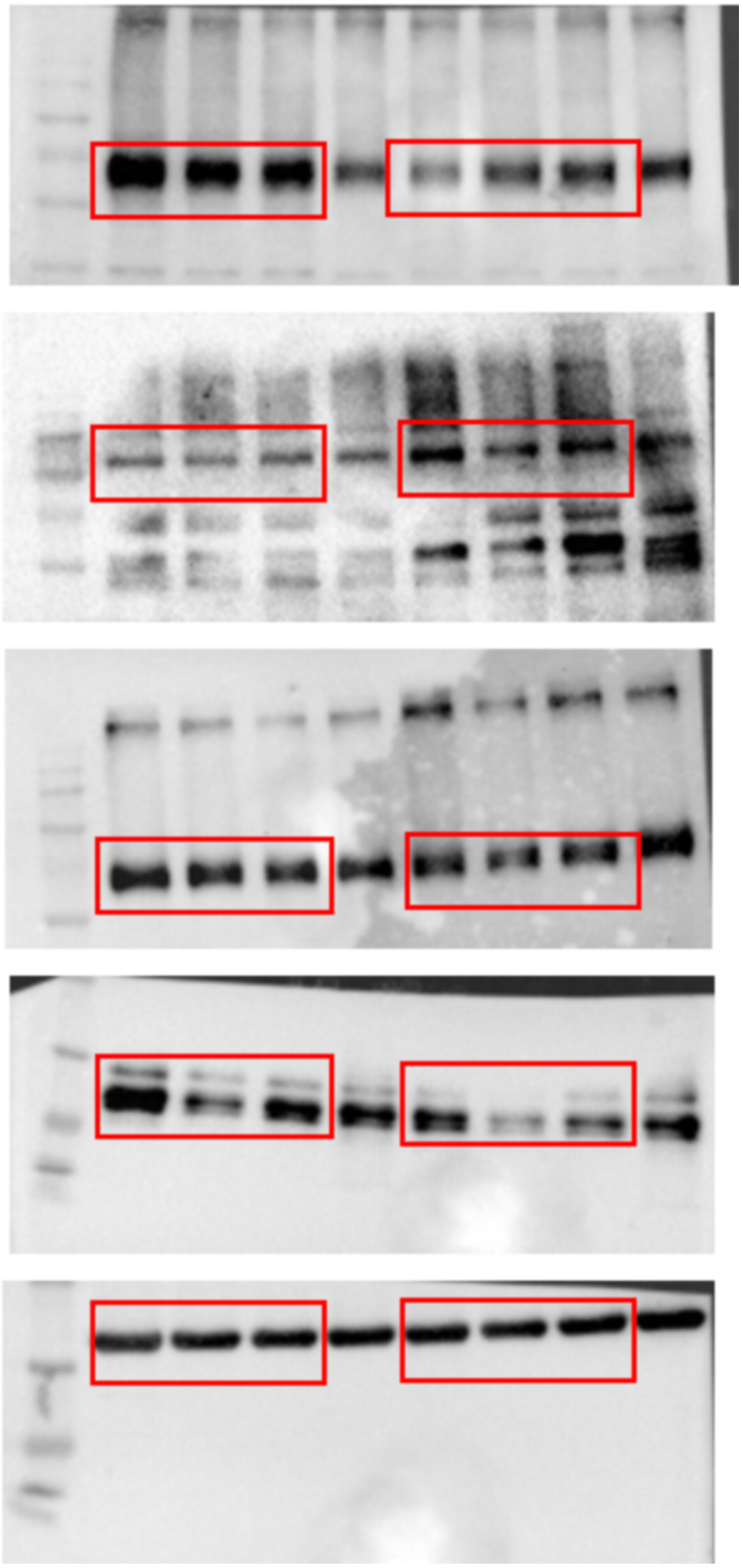

Journal of Cell Science • Supplementary information

Fig 6A

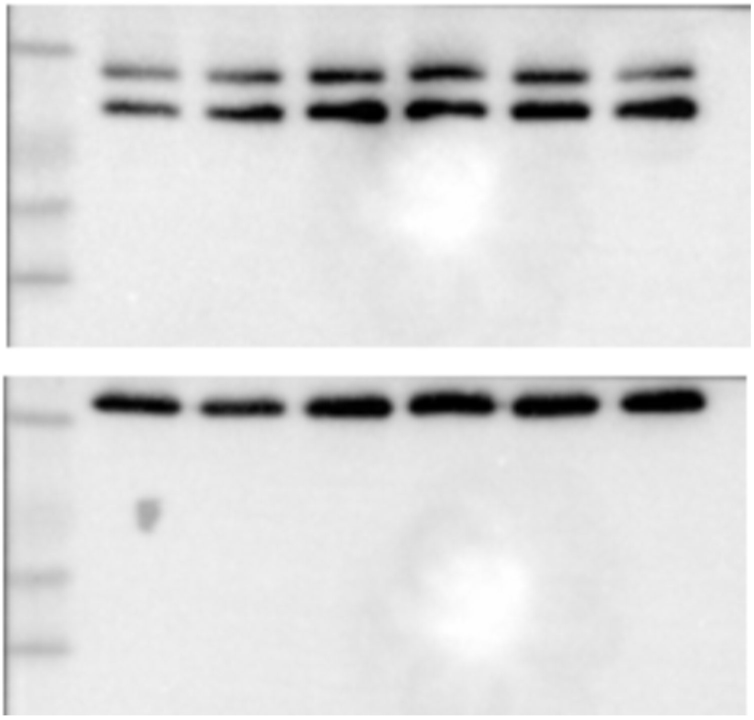

Journal of Cell Science • Supplementary information

Supplementary Figure 5A

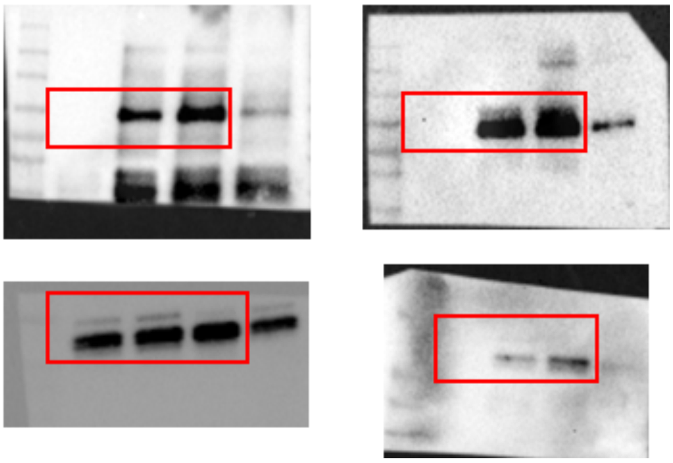

Supplementary Figure 6A

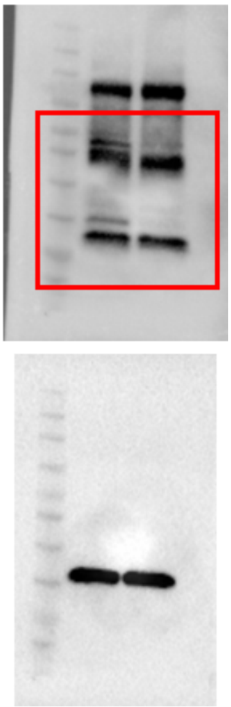

Supplementary Figure 6B

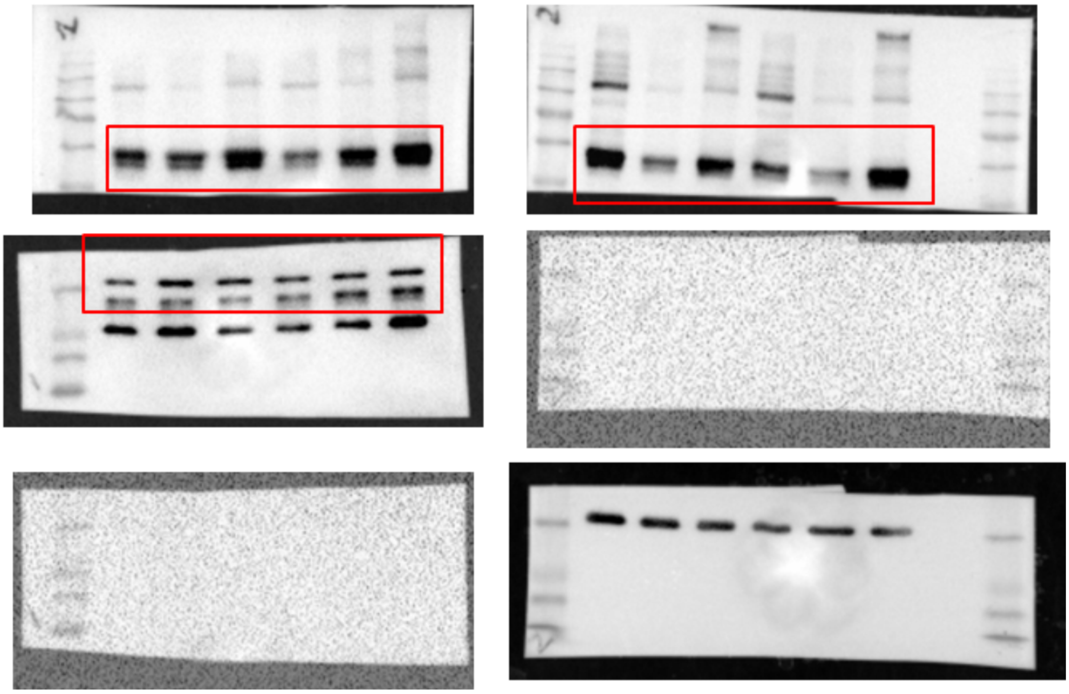

Fig. S7.

Journal of Cell Science • Supplementary information
